# Mechanism of right thoracic adolescent idiopathic scoliosis at risk for progression; a unifying pathway of development by normal growth and imbalance

**DOI:** 10.1186/s13013-015-0030-2

**Published:** 2015-01-27

**Authors:** Christian Wong

**Affiliations:** Fuglevadvej, 2800 Kgs, Lyngby, Denmark

## Abstract

Adolescent idiopathic scoliosis is regarded as a multifactorial disease and none of the many suggested causal etiologies have yet prevailed. I will suggest that adolescent idiopathic scoliosis has one common denominator, namely that initial curve development is mediated through one common normal physiological pathway of thoracic rotational instability. This is a consequence of gender specific natural growth of the passive structural components of thoracic spinal tissues for the adolescent female. This causes an unbalanced mechanical situation, which progresses if the paravertebral muscles cannot maintain spinal alignment. The alteration in the coronal plane with the lateral curve deformity is an uncoupling effect due to a culmination of a secondary, temporary sagittal plane thoracic flattening and of a primary, temporary transverse plane rotational instability for the adolescent female. Treatment of adolescent idiopathic scoliosis should address this physiological pathway and the overall treatment strategy is early intervention with strengthening of thoracic rotational stability for small curve adolescent idiopathic scoliosis.

## Introduction

Adolescent idiopathic scoliosis has been described as early as 400 BC by Hippocrates [1]. Since then, numerous studies have been conducted to clarify the etiologies behind adolescent idiopathic scoliosis, suggesting a broad variety of causes: central nervous system-related as a result of cortical brain development or disharmony in development between the somatic and autonomic nervous system, growth-related due to anterior spinal overgrowth or asymmetric rib growth, genetic as recognized from twin studies, hormonal related to melatonin, calmodulin or leptin system dysfunction, biomechanical as a result of a medullary or ligamental mechanical tether, related to asymmetry in pre-existing rotational patterns or developmental changes in the trunk, and many others causal theories [2-16]. The mosaic of these theories are now being brought together into a more coherent framework such as the double neuro-osseous theory and a multimodal causal treatment may emerge in the future [17,18]. Still, so far no single causal theory for adolescent idiopathic scoliosis has prevailed and it is still a conundrum and regarded as a multifactorial disease [2,3,18]. However, the enigma of the common clinical characteristics of adolescent idiopathic scoliosis with a right primary thoracic curve at risk of progression (AIS), developing in the adolescent girl during spinal growth, cry out for a unifying theory, clarifying these aspects, or as formulated by Weinstein; “the key question of how and why initial small curve develops have not been answered” [2,3,19,20]. This paper will suggest that AIS should not be explained by one unifying causal theory. The etiology, being by nature multifactorial, is initiated and mediated, however, through one common physiological mechanism of thoracic rotational instability (RI) in turn being a consequence of gender specific natural growth, causing an unbalanced mechanical situation. I will suggest that it is a common physiological pathway of the normal spinal growing tissue bring about or mediates the ‘etiologies’ of the progressive structural AIS, and it is the ‘mediating culprit’ that should be addressed when treating. The foundation for this physiological pathway has already been paved thoroughly in the scientific literature, but has to be seen in a pediatric view of growth with the biomechanical perspective of spinal instability in regards to treatment. Rather than considering it to be a coherent etiology accounting for all gender, age and curve variations, it should be seen as relevant observations leading to a suggested physiological pathway for scoliosis with a right primary thoracic curve, since treatment of these are required due to risk for progression [20]. Principally, such a common pathway to AIS should answer the following questions; why does AIS develop in adolescent girls? Why is the right thoracic main curve with rotation at risk of progression? And how to stop it?

## Hypothesis

Firstly, AIS seems to be uniquely human and have been attributed to the reclined erect posture of human bipedalism [3,19,21]. Secondly, early AIS is almost equally common in both genders, but the incidence and risk of progression increase for girls when reaching the growth spurt [2,20,22]. Thirdly, it seems, that the adolescent female (AF) with AIS is taller, but does not differ with respect to a faster growth velocity and maturation rate as believed earlier [3,22,23]. These circumstances imply that AIS is not due to abnormal growth but rather a consequence of normal development for the upright modern human and due to gender-related differences [19]. Changes during growth of supporting structural tissue of the spinal bones, ligaments, and muscles for the AF would therefore seem important for the initiation and development of AIS.

In principle, AIS has developed as a consequence of the spine growing into a mechanically unstable column and seems to progress in a self-sustaining ‘vicious cycle’ due to the effect of gravity and asymmetric loads in a growth-modulating buckling-like manner [3,19,24]. Principles of mechanical engineering or column stability tell us, that when a column structure is higher, as in the growing spine, it is less stable and would require stronger inherent constraints for stability [19,25]. The spinal growth spurt for both sexes coincides with adolescence, but AF distinguish themselves from males by a significantly increased and with an earlier thoracic growth, and developing ‘sexual dimorphism’ of slender vertebra [23,26,27]. The factors of thoracic growth, slender vertebrae, and reclined posture make the thoracic spine susceptible to rotational instability when subjected to the axial load of gravity - especially when subjected to dorsal shear loads as for humans [3,21,27,28]. These factors coincide with the development of a temporarily ‘straighter’ back in sagittal plane of the thoracic spine, creating a ‘lordotic spinal posture’ with a smaller thoracic kyphosis at the vertebral growth peak for the AF as observed by Adams as early as in 1882 [3,21,22,24,28-31]. This might be initiated in the thoracolumbar junction [12] or as a consequence of growth-induced changes in the lumbar lordosis, sacral slope and pelvic incidence [32], but it contributes to the overall RI, since it redirects the axial load of gravity dorsally onto the apices of the transversely heart-shaped thoracic vertebrae with ‘biplanar’ asymmetry [19,28,29]. The thoracic vertebrae will then perform a lateral bending when rotating, due to the ‘coupled motion’ between the thoracic vertebrae [33]. This mechanism interconnects rotation and lateral bending motion, whilst forcing the posterior element towards the concavity of the AIS [19,21,29,33-36]. The alteration in the coronal plane is therefore an uncoupling effect due to a culmination of a secondary sagittal plane deformity and of a primary transverse plane RI for the AF. The concept of axial rotational imbalance is not novel and has already been recognized as a key factor for AIS development [37,38]. The changed dorsal-directed load would affect the posterior elements of the facets and costovertebral joints [28]. This makes the thoracic frontally-oriented facet joints susceptible to RI [28,39,40]. The thoracic spine will follow the preexisting rotational pattern to the right, since the normal adolescent also has a right superior facet asymmetry [3,39-41]. Moreover, when the costovertebral joints’ mechanical integrity is compromised, it would seem to accentuate rotation of the vertebrae and subsequently the ‘coupled motion’ of lateral bending [42]. Yet another ‘coupled motion’ seems to occur, in which rotation of the vertebrae subsequently lead costae to rotate, thus probably facilitating the ‘hump’ [35,43]. Moreover, the thoracic cage increases in size during this period of spinal growth, whilst displaying truncal asymmetry and a relative narrowing for the AF, adding to the instability [12,44].

Changes in flexibility of the passive structures of spinal soft tissue including ligaments and discs occur for the normal AF. Spinal mobility develops with a significant increase in thoracic rotation to the right in adolescence, otherwise becoming stiffer in the thoracic spine in all other planes for the AF [41,45,46]. Lumbar lateral flexion also increases with a shift from left to right and the spine develops significantly less overall anterior flexion for the AF [41,47,48]. These gender-related changes in flexibility favor RI by developing a mechanical situation of increased lumbar lateral flexion and right thoracic rotation for the AF without the ability to compensate for the latter by flexing forward thoracically in the sagittal plane [19]. These factors force the spine into the classic shape of AIS under the aforementioned ‘right’ circumstances of increased growth, increased flexibility, and ‘lordotic’ posture, which peaks for girls in early adolescence. Not surprisingly the spine regains rotational stability as the AIS curve develops and progresses [49,50].

Already half a century ago muscle imbalance was suggested as causation for AIS [3,19,51,52]. Differences in paravertebral muscle morphology, electromyographic response, and behavioral response to exercise have indicated that muscle imbalance is a cause for progression or regression of AIS [2,3,52-55]. The majority of AIS remain stable, but the rest either regress or progress [52]. This has led to a muscle balancing/tuning theory, where the spinal muscles - in a heightened state - try to return the spine to a neutral position, displaying a ‘wavy’ curve pattern with fluctuations in lateral curve shape in mild and early AIS when followed closely [20,22,52]. The paravertebral muscles are suggested to have a correcting function, trying to straighten the thoracic spine as a compensatory mechanism to the increasing instability. The compensatory role of the paravertebral muscles is substantiated by the partial straightening of the spinal column of AIS during nighttime using electric muscle stimulation [56]. However, AIS progresses when the paravertebral muscles fail to compensate and stabilize for the inherently rotationally unstable thoracic spine [3,19,29].

The many aforementioned proposed etiological factors contribute to the instability, thus disturbing the spinal equilibrium and trigger initiation of AIS through the proposed physiological pathway. The anatomical variation, and the multiple and variable destabilizing factors give rise to the morphological variation in AIS curves. Morphological changes in spinal bone would seem to be an adaptive response and secondary to the initial thoracic rotational instability [29,40].

In conclusion, the obvious lateral spinal curvature in the coronal plane is a consequence rather than the cause of AIS, but “by the time the AIS achieve clinical significance, it is the secondary deformity which is obvious and masks or obscures the underlying primary deformity” [29]. Moreover, there is a physiological pathway for development of the classic right thoracic curve of AIS for AF, and this is right thoracic rotational instability. This described pathway should be addressed when treating AIS.

## Treatment

In the last decades the nature of treating the clubfoot has been changed successfully by Ignatius Ponseti, where his first article was hypothetical by nature as this one, and to paraphrase his ‘concluding words’ [57]:

“We are handicapped because of our ignorance of the primary causes of the deformity of AIS…The altered form of the lateral curvature is the result rather than the cause of the deformity…and the essence of this deformity of the spine consists in the twisting of the thoracic spine due to rotational instability by a temporary development of thoracic hypokyphosis…Treatment entails correction of the position of the thoracic sagittal curvature as well as the stretching of thoracic ligaments and strengthening rotational thoracic and lumbar muscles. The younger the girl, the easier these corrections are. Success requires a thorough knowledge of the deformity and of the functional anatomy of the spine”.

I will suggest that treatment of primary right thoracic idiopathic scoliosis should address the physiological pathway, namely by early identification and treatment of small curve AIS, strengthening thoracic rotational stability by exercise and creating an external hyperkyphosing thoracic posture by light bracing or by other means of sagittal plane hyperkyphotic and coronal plane central autocorrection. Rotational strengthening exercises should apply appropriate parts of the principles of targeted training with focus specifically on trunk control of the chest [17,58]. Earlier brace intervention than that which is presently practiced has recently been examined, and the strategy of early intervention is somewhat substantiated [59]. Likewise, brace treatment focusing on sagittal plane correction by thoracolumbar junction hyperlordosation have shown both initial and short-term promising results of coronal plane correction [60,61], and autocorrective training, which would address the central nervous system-induced asymmetric spinal development, have also been recognized as an effective treatment [17,62]. The perspective of treating the underlying etiological factors are attractive, but do not seem eminent, hence treatment aimed at this proposed physiological pathway of AIS would (almost readily) be feasible. However, Proof of validity of the proposed physiological pathway and treatment are interrelated and will first arise after implementation of treatment and documentation of a shift in the natural history of AIS. Large prospective longitudinal intervention studies with randomization to either the current regime or my proposed treatment would be needed [2].

The efficacy of the current conservative treatment of bracing have been questioned [63,64], and it has been claimed that omitting bracing is inconsequential [65] It has nevertheless been prescribed for half a century [66]. Recently, bracing has been rigorously tested scientifically, and it seems to minimize the risk of progression for AIS in respect of the threshold for surgery [67]. However, it is still being questioned in spite of this meticulous scientific effort [68]. Moreover, bracing is physically and emotionally strenuous for the AF with a correspondingly low compliance [69-71]. From this point of view, it would seem inappropriate not to look for alternate treatment strategies [72].

In conclusion, in this study I have suggested a physiological mechanism that gives a comprehensive explanation to the enigmas of AIS. It lies inherently in the nature of this developmental disease that long-term systematic scientific proof is needed to confirm the predictions. However, this is written to inspire future AIS research.
